# INTERGENERATIONAL SUPPORT EXCHANGES AND OLDER PARENTS' CARE RECEIPT AND EXPECTATIONS

**DOI:** 10.1093/geroni/igac059.485

**Published:** 2022-12-20

**Authors:** Cindy Bui, Kyungmin Kim, Karen Fingerman

**Affiliations:** University of Massachusetts Boston, Boston, Massachusetts, United States; Seoul National University, Seoul, Seoul-t'ukpyolsi, Republic of Korea; The University of Texas at Austin, Austin, Texas, United States

## Abstract

Distinguishing between support and care, this study investigated how different types of past support exchanges with children were associated with older parents’ care receipt and expectations. Older parents (N=190; Mage=79.98) reported on tangible, non-tangible, and childcare support exchanges with each of their adult children (N=709; Mage=52.69) in two waves of the Family Exchanges Study (2008 and 2013). Multilevel, within-family, logistic regression models were estimated. Parents with functional limitations more likely received care from children whom they received more tangible support from at the prior wave. Parents without current limitations more likely named children whom they previously provided childcare support to and received more tangible support from as their expected future caregiver. These findings emphasize continuity in the transition from receiving tangible support to receiving and expecting care from adult children. The importance of older parents’ childcare support given to adult children also highlights reciprocity in intergenerational care exchanges.

